# Bone Mineral Density in Sjögren Syndrome Patients with and Without Distal Renal Tubular Acidosis

**DOI:** 10.1007/s00223-016-0112-z

**Published:** 2016-02-12

**Authors:** Tim Both, M. Carola Zillikens, Ewout J. Hoorn, Robert Zietse, Jan A. M. van Laar, Virgil A. S. H. Dalm, Cornelia M. van Duijn, Marjan A. Versnel, Naomi I. Maria, P. Martin van Hagen, Paul L. A. van Daele

**Affiliations:** Division of Clinical Immunology, Department of Internal Medicine, PO Box 2040, Room D-419, 3000 CA Rotterdam, The Netherlands; Department of Endocrinology, Erasmus Medical Center, Rotterdam, The Netherlands; Department of Nephrology & Transplantation, Erasmus Medical Center, Rotterdam, The Netherlands; Department of Immunology, Erasmus Medical Center, Rotterdam, The Netherlands; Department of Epidemiology, Erasmus Medical Center, Rotterdam, The Netherlands

**Keywords:** Bone mineral density, Sjögren syndrome, Distal renal tubular acidosis, Hydroxychloroquine, DEXA scan

## Abstract

Primary Sjögren’s syndrome (pSS) can be complicated by distal renal tubular acidosis (dRTA), which may contribute to low bone mineral density (BMD). Our objective was to evaluate BMD in pSS patients with and without dRTA as compared with healthy controls. BMD of lumbar spine (LS) and femoral neck (FN) was measured in 54 pSS patients and 162 healthy age- and sex-matched controls by dual-energy X-ray absorptiometry (DXA). dRTA was defined as inability to reach urinary pH <5.3 after an ammonium chloride (NH_4_Cl) test. LS- and FN-BMD were significantly higher in pSS patients compared with controls (1.18 ± 0.21 g/cm^2^ for patients vs. 1.10 ± 0.18 g/cm^2^ for controls, *P* = 0.008 and 0.9 ± 0.16 g/cm^2^ for patients vs. 0.85 ± 0.13 g/cm^2^ for controls, *P* = 0.009, respectively). After adjustment for BMI and smoking, the LS- and FN-BMD remained significantly higher. Patients with dRTA (*N* = 15) did not have a significantly different LS- and FN-BMD compared with those without dRTA (*N* = 39) after adjustment for BMI, age, and gender. Thirty-seven (69 %) pSS patients were using hydroxychloroquine (HCQ). Unexpectedly, pSS patients had a significantly higher LS- and FN-BMD compared with healthy controls. Patients with dRTA had similar BMD compared with patients without dRTA. We postulate that an explanation for the higher BMD in pSS patients may be the frequent use of HCQ.

## Introduction

Sjögren syndrome (SS) is a prevalent chronic autoimmune disease characterized by impairment of exocrine glands and systemic manifestations, affecting between 1 and 3 % of the general population [1]. SS can be present alone (primary Sjögren syndrome (pSS)) or accompanied by other autoimmune diseases such as systemic lupus erythematosus (SLE) or rheumatoid arthritis (RA) and is then called secondary Sjögren syndrome (sSS) [2, 3]. The classical symptoms of SS include dryness of mouth (xerostomia), dryness of eyes (xerophthalmia) and less commonly dryness of pharynx, larynx, and/or vagina [4]. Extraglandular manifestations can be divided into general symptoms (e.g., fatigue, arthralgia, and myalgia) and in systemic manifestations [5]. Common systemic manifestations include renal tubular acidosis, non-erosive symmetrical arthritis, interstitial lung disease, peripheral polyneuropathy, autoimmune thyroiditis, and B cell lymphoma [6–11].

Distal renal tubular acidosis (dRTA) is one of the less well recognized complications. Recently, we reported a considerably high prevalence of dRTA in pSS [7]. dRTA is characterized by the inability of patients to lower urinary pH <5.3 due to a defect in the proton secreting machinery of the alpha-intercalated cells in the collecting ducts of the kidney [12]. Patients with dRTA have a non-anion gap metabolic acidosis with urinary pH ≥5.3.

The association between chronic metabolic acidosis and alteration in bone cell function has been demonstrated both in vitro and in vivo [13, 14]. During metabolic acidosis, there appears to be an exchange of protons for calcium ions in bone mineral to buffer the excess of protons [13]. The metabolic effects of dRTA in pSS remain conflicting. Several case-series have shown a low bone mineral density (BMD) in patients with dRTA in pSS [15, 16]. Some recent studies report an increased prevalence of low BMD in patients with dRTA [17, 18], while other studies did not report a significant difference in patients with dRTA [19, 20]. Epidemiologic data on BMD in SS are lacking.

We hypothesized that BMD is significantly decreased in patients with pSS and especially in those with dRTA. Therefore, our aim was to evaluate BMD in pSS patients with and without dRTA as compared with healthy controls.

## Methods

### Study Cohort

Patients were selected from the outpatient clinic of the department of internal medicine (division of clinical immunology) of Erasmus MC in Rotterdam, The Netherlands. pSS was defined according to the Revised American-European classification criteria [21]. The results of salivary gland biopsy were retrieved when available. Additional inclusion criteria for this study included age >18 years and an estimated glomerular filtration rate >30 ml/min. The exclusion criteria were other underlying autoimmune diseases, known risk factors for osteoporosis (vitamin D level <20 nmol/l, untreated hyperthyroidism, hyperparathyroidism, use of corticosteroids (prednisone equivalent of >7.5 mg for >3 months in the last year), use of bisphosphonates, multiple myeloma, mastocytosis). All participants were asked about menopausal status (if applicable), current smoking and history of fractures, and use of medication (Table 1). Data from BMD in the healthy control group were obtained from the ERF (Erasmus Rucphen Family) study database [22]. The matching criteria for the controls were age and sex. For every pSS patient, three controls were selected.

**Table 1:** Characteristics of the study cohort

	Study cohort (*N* = 54)	No dRTA (*N* = 39)	dRTA (*N* = 15)	Control group (*N* = 162)
Demographics				
Age, years ± SD	57.3 ± 10.6	60.1 ± 9.4	50.2 ± 10.5	57.3 ± 10.6
Female gender, *n* (%)	50 (93)	36 (92)	14 (93)	150 (93)
Body mass index, kg/m^2^ ± SD	26.8 ± 6.2	26.9 ± 6.2	26.6 ± 6.6	27.3 ± 5.3
Current smokers, *n* (%)	2 (4)	0 (0)	2 (13)	60 (37)
Postmenopausal, *n* (%)	36/50 (72)	5/36 (14)	5/14 (36)	119 (69)
Age at menopause, years ± SD	47.4 ± 6.1	47.0 ± 6.5	48.4 ± 4.9	47.9 ± 6.1
Previous fractures, *n* (%)^§^	19 (35)	11 (28)	8 (53)	n.a
Disease duration, years ± SD	12.1 ± 7.2	12.2 ± 8.0	11.5 ± 5.6	–
Biochemical				
Serum 25-OH-Vitamin D, nmol/L ± SD	70.4 ± 21,8	69.7 ± 22.1	72.5 ± 21.7	
Serum intact PTH, pmol/L ± SD	4.3 ± 1.5	4.5 ± 1.5	3.7 ± 1.5	
Serum calcium, mmol/L ± SD	2.39 ± 0.08	2.39 ± 0.07	2.38 ± 0.16	
Serum phosphate, mmol/L ± SD	1.09 ± 0.15	1.10 ± 0.14	1.06 ± 0.16	
Serum creatinine, µmol/L ± SD	76.8 ± 18.6	72.9 ± 11.8	86.7 ± 27.9	
Serum PINP, µg/L ± SD	38.6 ± 18.5	41 ± 19.2	32.5 ± 15.4	
Serum BAP, µg/L ± SD	14.2 ± 4.1	14.7 ± 4.3	12.8 ± 3.3	
Serum NTX, nM BCE ± SD	17.2 ± 4.4	17.3 ± 4.6	16.8 ± 3.8	
Immunology				
Anti-nuclear antibodies, *n* (%)	41 (76)	28 (72)	13 (87)	
Rheumatoid factor, *n* (%)	32/42 (76)	21/29 (72)	11/13 (85)	
SSA/Ro52, *n* (%)	42 (78)	29 (74)	13 (87)	
SSA/Ro60, *n* (%)	40 (74)	26 (67)	14 (93)	
SSB/La, *n* (%)	31 (57)	18 (46)	13 (87)	
Positive salivary gland biopsy, *n* (%)^¶^	23/27 (85)	14/18 (78)	9/9 (100)	
Medication				
Hydroxychloroquine, *n* (%)	35 (69)	23 (59)	12 (80)	
Vitamin D supplements, *n* (%)	11 (20)	9 (23)	2 (13)	
Glucocorticoids, *n* (%)	3 (6)	2 (5)	1 (7)	
Other immunosuppressive drugs, *n* (%)^†^	4 (7)	2 (5)	2 (13)	

Data are presented as mean ± standard deviation (SD) and no. (%)

^¶^ Salivary gland biopsies were performed in 34/54 patients, but a focus score could be retrieved for only 27 patients; Rheumatoid factor was measured in 42/54 patients

^†^ Other immunosuppressive therapy consisted of azathioprine, colchicine, or methotrexate

^§^ Data about previous fractures could not accurately be retrieved

### Bone Mineral Density

In all subjects, we measured BMD of the lumbar spine (L2-L4) and femoral neck using a dual-energy X-ray absorptiometry (DXA) scanner (Prodigy Pro Full P8, enCORE™ Software Platform, GE Medical Systems Lunar, Belgium). Scans were performed according to the manufacturer’s guidelines and analyzed according to ISCD rules [23]. The healthy control group was scanned with a different DXA device from the same type (GE Lunar Prodigy device, GE Healthcare, USA) [24]. As described by Enneman et al., a cross-calibration was performed using a spine phantom which showed that the measurements of the new scanner (the one we used for the patients in the current study) were slightly higher by a factor 1.0101 [24]. Therefore, we divided our results by this factor for comparison with the data from the ERF study. BMD was expressed in grams per square centimeters.

### Biochemical Parameters

In all patients, we measured vitamin D status. Anti-nuclear antibodies (ANA), SSA/Ro52, SSA/Ro60, SSB/La auto-antibodies, and rheumatoid factor (RF) were also measured in all patients using previously reported methods [25]. Serum was collected before 10:00 a.m. and analyzed the same day. Patients were not instructed to be fasting. The following bone turnover markers (BTMs) were measured in patients: serum N-terminal propeptide of type I procollagen (PINP) and serum bone-specific alkaline phosphatase (BAP, both as measures of bone formation) and serum N-terminal crosslinking telopeptide of type I collagen (NTX, as measure for bone resorption). There were no data available on BTMs in the healthy control group.

### Distal Renal Tubular Acidosis

dRTA was defined as an abnormal NH_4_CL test and the absence of any other known causes for dRTA (e.g., medication, hypercalciuria) [12]. The NH_4_CL test is defined as abnormal if patients fail to achieve a urinary pH <5.3 within 4 h after intake of ammonium chloride (1 ml/kg body weight) [26].

### Statistics

All results are expressed as means with standard deviations. Comparisons of the normally distributed continuous variables between two groups were performed using the student *T* test. Since BTMs were not normally distributed, we compared BTMs between the two groups using the Mann–Whitney *U* test. Linear regression analysis was used to estimate the effect of having pSS on BMD before and after adjustment for body mass index (BMI) and smoking. Linear regression analysis was used to estimate the effect of having dRTA on BMD before and after adjustment for BMI, age, and gender. A *P* value <0.05 was considered significant. All analyses were performed in SPSS (version 21, IBM).

## Results

### Study Cohort and Baseline Characteristics

The study cohort included 54 patients with pSS and the control group consisted of 162 subjects. Initially, 62 patients participated in the study. Eight patients were excluded, including four patients who were unable to complete the NH_4_CL test due to repeated vomiting, one patient in whom the DXA-scan was not reliable due to scoliosis and three patients were using bisphosphonates. No patients were excluded because of long-term use of corticosteroids and only three patients were using low dose corticosteroids for a medical condition other than pSS. The baseline characteristics of the study cohort are shown in (Table 1). Similar to previous studies on pSS, our cohort has a female:male ratio of approximately 10:1 [27]. Thirty-seven (69 %) patients with pSS were using HCQ. In addition to medication for pSS, other commonly used drugs in this cohort were vitamin D with calcium supplements (*N* = 11). None of these patients reported a fracture in their medical history.

### BMD of pSS Patients Compared with Healthy Controls

**Fig. 1:**
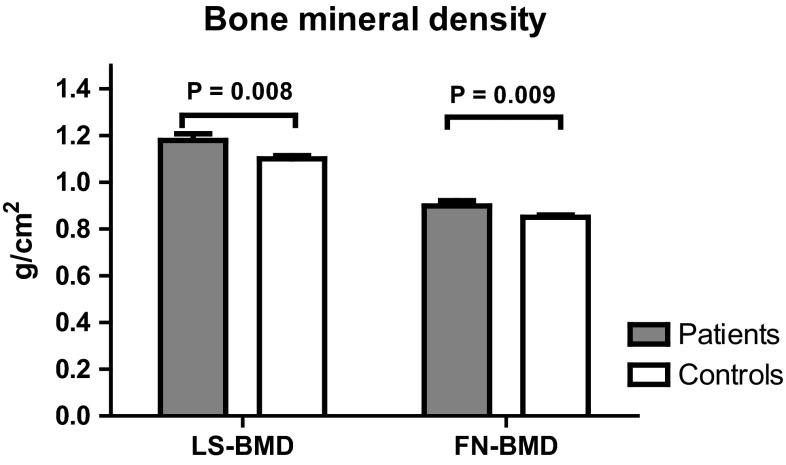
Comparison of BMD between patients and controls. The graph shows the bone mineral density of the lumbar spine and the femoral neck of patients with primary Sjögren syndrome (*N* = 54) compared with healthy age- and sex-matched controls (*N* = 162). *SD* standard deviation, *BMD* bone mineral density, *LS-BMD* bone mineral density of the lumbar spine, *FN-BMD* bone mineral density of the femoral neck

BMD of fifty-four pSS patients was compared with the age- and sex-matched control group of 162 subjects. The LS- and FN-BMD were significantly higher in the pSS patients compared with the healthy control group (1.18 ± 0.21 g/cm^2^ for pSS patients vs. 1.10 ± 0.18 g/cm^2^ for the control group, *P* = 0.008 and 0.9 ± 0.16 g/cm^2^ for pSS patients vs. 0.85 ± 0.13 g/cm^2^ for the control group, *P* = 0.009, respectively) **(**Fig. 1**)**.

After adjustment for BMI and smoking, the LS- and FN-BMD remained significantly higher (*β* = 0.10 ± 0.030 g/cm^2^, *P* < 0.001 and *β* = 0.077 ± 0.022 g/cm^2^, *P* < 0.001) **(**Table 2**)**.

**Table 2:** Multiple regression analysis of factors related to LS- and FN-BMD between patients (*N* = 54) versus controls (*N* = 162)

Variable	LS-BMD			FN-BMD		
	B	Std. error	*β*	*B*	Std. error	*β*
(Constant)	0.797	0.063		0.587	0.047	
pSS	0.103	0.030	0.237**	0.077	0.022	0.235**
BMI	0.010	0.002	0.298**	0.009	0.002	0.350**
Smoking	0.061	0.029	0.147*	0.045	0.021	0.143*

*Std. error* standard error of the mean, *pSS* primary Sjögren syndrome, *BMI* body mass index, *LS-BMD* bone mineral density of the lumbar spine, *FN-BMD*, bone mineral density of the femoral neck

* *P* 0.01 < 0.05

** *P* < 0.01

### The Effect of Distal Renal Tubular Acidosis on BMD

Fifteen pSS patients had an urinary acidification defect as measured by the NH_4_CL test. Between both groups the levels of biochemical parameters and the use of medication were similar **(**Table 1**)**. Patients with dRTA were significantly younger compared with those without dRTA **(**Table 1**)**. Both the LS- and FN-BMD were significantly higher in patients with an urinary acidification defect compared with those without an urinary acidification defect (LS: 1.29 ± 0.16 g/cm^2^ vs. 1.14 ± 0.21 g/cm^2^, *P* = 0.018 and FN: 1.0 ± 0.19 g/cm^2^ vs. 0.87 ± 0.14 g/cm^2^, *P* = 0.007). After adjustment for BMI, age, and gender, both the LS- and FN-BMD were not significantly higher anymore (LS: *β* = 0.12 ± 0.064 g/cm^2^, *P* = 0.065 and FN: *β* = 0.07 ± 0.049 g/cm^2^, *P* = 0.16) **(**Table 3**)**.

**Table 3:** Multiple regression analysis of factors related to LS- and FN-BMD between patients with dRTA (*N* = 15) versus patients without dRTA (*N* = 39)

Variable	LS-BMD			FN-BMD		
	*B*	Std. error	*β*	*B*	Std. error	*β*
(Constant)	1.292	0.217		1.175	0.166	
dRTA	0.121	0.064	0.264	0.070	0.049	0.184
BMI	0.007	0.004	0.220	0.003	0.003	0.113
Gender	−0.177	0.100	−0.226	−0.008	0.076	−0.012
Age	−0.003	0.003	−0.157	−0.006	0.002	−0.405*

*Std. error* standard error of the mean, *dRTA* distal renal tubular acidosis, *BMI* body mass index, *LS-BMD* bone mineral density of the lumbar spine, *FN-BMD*, bone mineral density of the femoral neck

* *P* < 0.01

### Bone Turnover Markers in pSS Patients

In patients with dRTA serum, PINP was not significantly higher compared with patients without dRTA (*P* = 0.093). The other marker for bone formation, BAP, was also not significantly different between both groups (*P* = 0.11). The bone resorption marker NTX was not significantly different between patients with and without dRTA (*P* = 0.92) **(**data not shown**)**.

## Discussion

In the present study, we found that, contrary to expected, pSS patients have significantly higher BMD than healthy age- and sex-matched controls. We searched the available literature but did not find another study reporting BMD measurements in pSS patients as compared with a healthy control group. Studies concerning BMD in autoimmune diseases are mainly performed in lupus patients. In agreement with Arampatzis et al. and Pongchaiyakul et al., we found that patients with an urinary acidification defect did not have a significantly different LS- and FN-BMD compared with those patients without an urinary acidification defect [19, 20].

Bushinsky et al. reported a decreased bone mineralization in an acidotic environment in both in vitro and in vivo studies [13, 14]. This makes us wonder what the reason is that we did not find a lower BMD in patients with dRTA.

We hypothesize that the observed BMD in pSS patients may be related to the use of hydroxychloroquine (HCQ) which the majority (69 %) of patients in our study was using. In case of systemic manifestations, therapy with non-steroidal anti-inflammatory drugs or HCQ is advised. HCQ has proven to be effective against fatigue, arthralgia, and myalgia [28, 29]. Lakshminarayanan et al. and Mok et al. reported that in lupus the use of HCQ was associated with increased BMD of the hip [30, 31]. In both studies, disease activity and use of corticosteroids were not significantly different between both groups. Additionally, Xiu et al. recently reported a reduced osteoclastogenesis by TRAF3 degradation due to the effects of chloroquine in mice, which may suggest that HCQ has direct effects on bone metabolism [32]. Based on these clinical and biochemical studies, we hypothesize that HCQ may have beneficial effects on BMD.

In our cohort, it is unknown how long these patients were treated with HCQ. We also did not have information about past use of HCQ in patients, who are not using it currently. Therefore, analyzing a possible association between HCQ use and BMD would not be reliable in our cohort. To demonstrate whether the use of HCQ has beneficial effects on human bone cells, in vitro studies should be performed.

We analyzed whether patients with dRTA also had different BTM measurements compared with patients without an urinary acidification defect. Since patients with dRTA had similar LS- and FN-BMD compared with those without dRTA, we expected that the BTMs measurements would not be significantly different between both groups. Indeed, all three BTMs (PINP, NTX, and BAP) were not significantly different between patients with and without an urinary acidification defect. Unfortunately, we could not compare BTM measurements between pSS patients and the healthy control group since data about BTM measurements in the healthy controls is lacking.

The strength of this study is that we have reported new data about the BMD values in a large cohort of pSS patients. In addition, we analyzed the effects of dRTA, a common complication of pSS, on BMD in pSS patients. A limitation of this study is that we used a different DXA scanner compared to Zillikens et al. although the type of machine was the same and calibration was performed with a spine phantom, making this an unlikely explanation for our findings [22].

In conclusion, we found that both the LS- and FN-BMD were higher in patients with pSS than in age and sex-matched healthy controls. In addition, LS- and FN-BMD in patients with an urinary acidification defect is comparable with patients without an urinary acidification defect. An explanation for the high BMD in pSS patients may be the frequent use of HCQ, but future studies will have to confirm whether indeed use of HCQ is associated with higher BMD.
